# Rhabdomyolysis associated with single-dose intravenous esomeprazole administration: A case report

**DOI:** 10.1097/MD.0000000000004313

**Published:** 2016-07-22

**Authors:** Dae-Hong Jeon, Yire Kim, Min Jeong Kim, Hyun Seop Cho, Eun Jin Bae, Se-Ho Chang, Dong Jun Park

**Affiliations:** a Department of Internal Medicine, Gyeongsang National University Hospital, Jinju; b Department of Internal Medicine, Changwon Gyeongsang National University Hospital, Changwon; c Department of Internal Medicine, Gyeongsang National University School of Medicine, Internal Medicine; d Institute of Health Science, Gyeongsang National University, Jinju, South Korea.

**Keywords:** acute kidney injury, proton pump inhibitor, rhabdomyolysis

## Abstract

**Background:**

Proton pump inhibitors are usually safe, although serious adverse effects can occur. We report the first case of rhabdomyolysis associated with single-dose intravenous esomeprozole administration.

**Methods:**

A 45-year-old Korean male visited our emergency room because of persistent lower chest discomfort that started 10 hours before. He had been diagnosed with diabetes and coronary heart disease, but discontinued oral hypoglycemic agents 1 month earlier. He continued to take medications for coronary heart disease. There was no abnormality on an electrocardiogram or in cardiac enzymes. Initial laboratory findings did not show abnormalities for muscle enzymes. Esomeprozole 40 mg was administrated intravenously for the control of his ambiguous chest discomfort. Then, 12 hours later, he complained of abrupt severe right buttock pain. An area of tender muscle swelling 8 cm in diameter was seen on his right buttock area. Creatine kinase and lactate dehydrogenase were elevated to 40,538 and 1326 U/L, respectively. A bone scan using 20 mCi of ^99m^Tc-hydroxymethylene diphosphonate was compatible with rhabdomyolysis.

**Results:**

His muscular symptoms, signs, and laboratory findings improved markedly with conservative management, including hydration and urine alkalinization. He is being followed in the outpatient department with no evidence of recurrence.

**Conclusion:**

We should keep in mind that single-dose intravenous administration of esomeprazole can induce rhabdomyolysis.

## 1 Introduction

Esomeprazole, the (*S*)-isomer of omeprazole,^[1,2]^ is available in both oral and intravenous formulations; it is usually indicated for the treatment of gastroesophageal reflux disease (GERD), *Helicobacter pylori* eradication, and the prevention of nonsteroidal anti-inflammatory drug induced gastric ulcers.^[3–5]^ Numerous clinical trials including many patients have demonstrated that esomeprazole is tolerable and safe for both short- and long-term use.^[6–8]^ Esomeprazole, like other proton pump inhibitors (PPIs), has few adverse effects, with reported rates of adverse effects ranging from 1% to 5%. The most common side effects include headache, diarrhea, abdominal pain, and nausea. These adverse effects, excluding diarrhea, are usually not related with age, dosage, or duration of treatment. Mean changes in laboratory measurements due to esomeprazole are usually small and not clinically meaningful.^[5]^ In addition, esomeprazole did not cause clinically significant electrocardiographic (ECG) changes.^[9]^

Rhabdomyolysis is induced by skeletal muscle breakdown, leading to the leakage of muscle cell contents, such as myoglobin, electrolytes, and other sarcoplasmic proteins, into the circulation.^[10,11]^ Acute kidney injury (AKI) complicated by rhabdomyolysis is quite common, representing about 7% to 10% of all cases of AKI in the United States.^[12,13]^ Eight categories of events are well known to provoke rhabdomyolysis: trauma, exertion, muscle hypoxia, genetic defects, infections, body temperature changes, metabolic and electrolyte disorders, and drugs and toxins.^[11,12,14]^ Rhabdomyolysis associated with PPIs has been reported sporadically.^[15–17]^ However, there has been no report of a PPI associated with rhabdomyolysis after single-dose intravenous administration. Thus, here we report the first case of rhabdomyolysis occurring after single-dose intravenous esomeprazole administration.

## 2 Case presentation

A 45-year-old male patient visited the emergency room (ER) because of lower chest discomfort starting 6 hours earlier. He also complained of thirst and limb numbness. His medical history included diabetes mellitus, bronchial asthma, and unstable angina for 4 years, 2 years, and 1 year, respectively. He had been taking aspirin (100 mg/d), clopidogrel (75 mg/d), atorvastatin (10 mg/d), and candesartan (8 mg/d) for 1 year without changes. He took those medicines on the morning of his admission. He regularly used a Symbicort Turbuhaler® (AstraZeneca Korea, Seoul, Republic of Korea) (160/4.5 μg) for asthma management. One month before the present admission, he discontinued the oral hypoglycemic agents prescribed by his primary physician. He denied recent alcohol consumption and had quit smoking 1 year earlier. Except for these symptoms, he initially had no complaints, such as muscular pain, fever, upper respiratory symptoms, or signs, on visiting the ER. He had no history of excessive physical activity or recent trauma.

On initial physical examination in the ER, his vital signs were as follows: blood pressure, 100/60 mm Hg; heart rate, 71 beats/min; respiratory rate, 20 times/min; and body temperature, 36.6 °C. On chest auscultation, no abnormal sounds, such as rales or wheezing, were audible, and his heartbeat was regular without murmur. His general skin turgor was decreased, and his tongue was dry. His conjunctivae were not anemic and sclerae were not icteric. There were no palpable lymph nodes in the head or neck area. Organomegaly was not seen in the abdomen. No pretibial pitting edema, muscular swelling, or skin color changes were detected on either lower extremity. There was no tenderness in the upper or lower extremities. His muscle power was within normal limits.

Compared with a previous ECG, the rhythm and voltage were unchanged. His initial troponin-I was less than 0.1 ng/mL, creatinine kinase (CK) was 144 U/L (0–190 U/L), lactate dehydrogenase (LDH) was 220 U/L (135–225 U/L), and CK-MB, subunit of CK, was 3.5 ng/mL. Other initial laboratory data were hematocrit 45% (39%–52%), hemoglobin 14.5 g/dL (13–17 g/dL), white blood cell count 11,280/mm^3^ (4000–10,000/mm^3^, neutrophils: 68.2%, lymphoid cells: 25.6%, and monocytes: 4.5%), and platelet count 256,000/mm^3^ (130,000–400,000/mm^3^). Liver function tests were as follows: alkaline phosphatase 84 IU/L (35–130 IU/L), aspartate aminotransferase (AST) 21 IU/L (0–37 IU/L), alanine aminotransferase (ALT) 42 IU/L (0–41 IU/L), and glucose 696 mg/dL. Hemoglobin A1c (HbA1c) was 9.3% (4.2%–5.9%). Blood urea nitrogen (BUN) and creatinine were 19.9 mg/dL (6.0–20.0 mg/dL) and 1.11 mg/dL (0.6–1.2 mg/dL), respectively. Electrolyte results were sodium 123 mmol/L (135–145 mmol/L), potassium 4.7 mmol/L (3.3–5.1 mmol/L), and chloride 80 mmol/L (98–110 mmol/L). Serum osmolality was 296 mOsm/kg. Urinalysis with micro revealed no protein or blood on a dipstick and red blood cell 1 to 4/high-power field.

**Figure 1:**
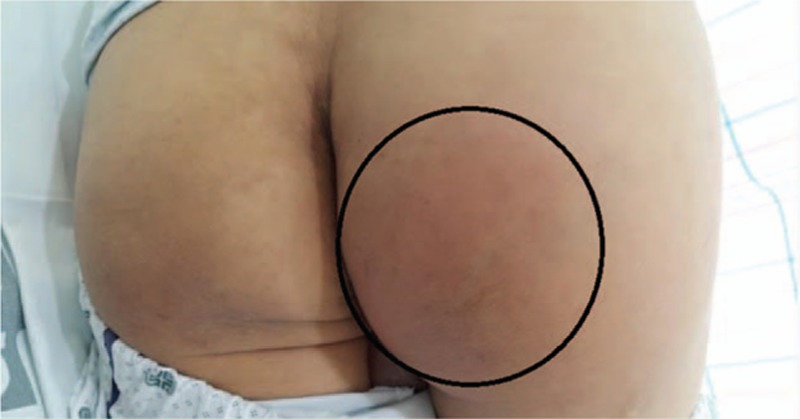
Red and swollen right buttock which is 8 cm in diameter (encircled area).

**Figure 2:**
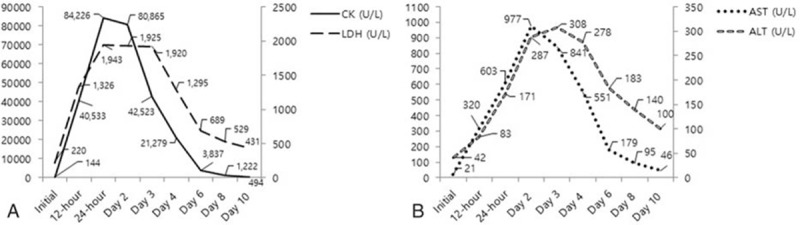
(A) and (B) The change of serum creatinine kinase, lactate dehydrogenase, aspartate aminotransferase, and alanine aminotransferase.

**Figure 3:**
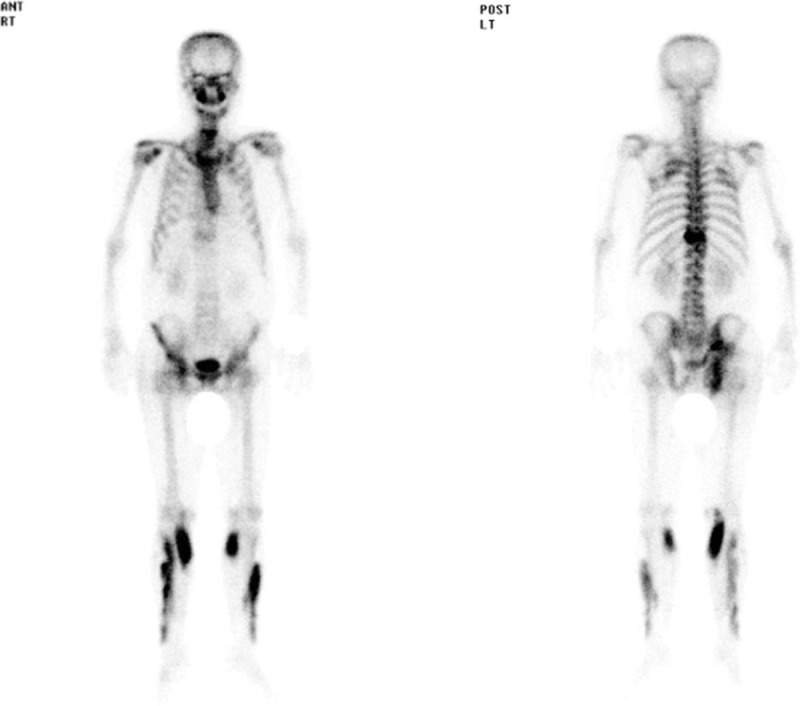
Bone scan showing multiple uptakes in both tibia and fibula and right buttock area.

Continuous intravenous insulin and hydration with normal saline were started. In addition, 40 mg of esomeprazole was infused intravenously under a suspicion of GERD. Glucose levels, measured by a blood sugar meter, decreased to 240 mg/dL and chest discomfort improved 6 hours after this management. In the mean time, there were no other oral, intramuscular, and intravenous drug therapies except for oral lactulose for his constipation. He felt mild discomfort in the right buttock at that time but did not complain to the medical team. However, he complained of severe right buttock pain 12 hours after the esomeprazole infusion. Tender muscle swelling of 8 cm in diameter was seen in the right buttock area and a reddish skin color change was noted (Fig. 1). Serum CK and LDH levels increased to 40,533 and 1326 U/L, respectively, and AST and ALT levels also increased to 320 and 83 IU/L, respectively. BUN and creatinine levels were 23.9 and 1.49 mg/dL, respectively. We started vigorous hydration through isotonic saline infusion and urine alkalinization by intravenous bicarbonate infusion. Another 40 mg of esomeprazole was administered because we did not regard esomeprazole as the causative agent of the rhabdomyolysis at that time. Serum CK, LDH, AST, and ALT levels were 84,226, 1943, 603, and 171 IU/L, respectively, 24 hours later. On the third day in hospital, he was admitted to the nephrology ward. Esomeprazole was ended, and hydration and alkalinization were continued. Serum CK and LDH levels decreased gradually (Fig. 2A and B), and his muscular symptoms and swelling in the buttock area improved gradually. A bone scan using 20 mCi of ^99m^Tc-hydroxymethylene diphosphonate was performed on the seventh day in hospital and revealed multiple and diffuse uptake in the soft tissues of the right buttock and both lower legs (Fig. 3). His clinical manifestations and laboratory findings improved, so he was discharged on the 12th day. We added oral hypoglycemic agents, linagliptin 5 mg and metformin 500 mg, to his previous drugs (aspirin, clopidogrel, candesartan, and atorvastatin). He is being followed in our outpatient department with no recurrence of rhabdomyolysis at 6 months.

## 3 Discussion

We report here the first case of rhabdomyolysis associated with single-dose esomeprazole intravenous administration. Our patient initially did not show clinical or laboratory evidence of rhabdomyolysis before esomeprazole administration in the ER. He was still in the ER when muscular pain and swelling presented after esomeprazole administration, but we did not suspect a relationship between rhabdomyolysis and esomeprazole, leading to administration of a second esomeprazole dose on the second day. We finally confirmed that esomeprazole was associated with rhabdomyolysis and ended the infusion of esomeprazole after he moved to the ward.

From the case reports in VigiBase and in the literature, Clark and Strandell^[18]^ reported 35 cases of rhabdomyolysis associated with PPIs in the literature, a serious reaction to the drugs. They also showed that a statin was taken concomitantly in 12 cases. Other drugs, such as lovastatin, amlodipine, and diazepam, were also taken, although no dates were given. Rhabdomyolysis occurred within the first week in 9 cases, between after 14 days and 3 months of treatment in 4 cases, and between 1.5 and 10 years in 3 additional cases. Time to onset was unknown in 19 cases. However, there are limitations to the report: VigiBase reports are not homogenous with respect to origin or to the probability that a pharmaceutical product caused the adverse reaction. Thus, no pure causal relationship between rhabdomyolysis and PPIs was evident. In our case, the causal relationship seems particularly prominent, in that esomeprazole was intravenously administered without other concomitant medicines except for intravenous insulin in the ER, and muscular symptoms and laboratory changes were confirmed immediately. We think that the administration of insulin is very unlikely to be one of the reasons for the rhabdomyolysis.

From PubMed research, 2 cases of rhabdomyolysis associated with esomeprazole have been reported.^[19,20]^ One case occurred in a patient with chronic heart failure and the other was in a patient taking atorvastatin and clarithromycin. The former patient also underwent aortic valve replacement and coronary artery bypass surgery and was administered several other medications including propofol, sufentanil, furosemide, amiodarone, meropenem, linezolid, and cathecholamines. Esomeprazole was administered intravenously (40–80 mg/d) for 1 month. They proposed that the dose of esomeprazole was correlated with rhabdomyolysis.^[19]^ However, his serum CK level was not elevated (although his serum myoglobin level was), but they did not mention the discordance between CK and myoglobin levels. Furthermore, there seemed to be no prominent correlation between esomeprazole withdrawal and restoration of myoglobin levels. The latter patient had a long medication history of atorvastatin (>1 year), a 6-week history of esomeprazole, and received 3 500-mg doses of clarithromycin just before admission.^[20]^ Thus, the rhabdomyolysis seemed not to be associated with esomeprazole, but with clarithromycin.

Most PPIs are metabolized to varying degrees by the hepatic cytochrome P450 enzymatic system, and drug metabolism may be altered by induction or inhibition of the cytochrome P enzymes.^[21]^ According to in vitro studies, esomeprazole was a weak inhibitor of cytochrome P450 (CYP3A4, -2B6, -2D6, -2C9, -2C8, and -1A2), but a potent inhibitor of CYP2C19 in human liver microsomes, compared with other PPIs, and esomeprazole might not reach the plasma concentrations needed to inhibit CYP3A4 in the normal clinical setting.^[22]^ Andersen et al extensively reviewed the pharmacokinetic drug interaction potential of esomeprazole. They showed that esomeprazole did not have clinically relevant interactions with 3A4 substrates, such as quinidine and clarithromycin, or the 2C9 substrate, (*S*)-warfarin. However, esomeprazole had different interaction with 2C19 substrates including (*R*)-warfarin, diazepam, and phenytoin.^[22]^

One report showed that 3- or 30-minute intravenous esomeprazole (40 mg) provides similar levels of intragastric acid control to the oral formulation,^[23,24]^ although little is known about the pharmacokinetic profile of esomeprazole after intravenous administration (whereas that of the oral formulation is well characterized).^[1,2]^ Niazi et al^[25]^ demonstrated that a higher infusion rate of intravenous esomeprazole showed higher *C*_max_ values, but did not affect other values such as the plasma concentration–time curve, plasma elimination half-life (*t*_1/2_), or plasma clearance in healthy volunteers. A previous study also showed that *C*_max_ values were higher after intravenous dosing than after oral dosing.^[26]^ Although the exact mechanism of rhabdomyolysis in our patient with a single-dose injection remains to be determined, an abrupt increase in *C*_max_ after intravenous infusion and drugs, which the patient had taken, might be associated with an elevation of the plasma level of esomeprazole, which might in turn be correlated with toxicity. Other possibility includes an idiosyncratic drug reaction, leading to some myopathy in the way that toxicity may occur after only single-dose use. Lastly, direct toxicity to muscle might be one of the mechanisms that can’t be ignored.^[27]^

In conclusion, physicians should keep in mind that a single-dose infusion of esomeprazole might be associated with rhabdomyolysis, directly or indirectly. Early recognition and intervention is important to reduce complications.
